# Some Aspects of the Physiology of the *Nyctotherus velox*, a Commensal Ciliated Protozoon Taken from the Hindgut of the Tropical Millipede *Archispirostreptus gigas*

**DOI:** 10.3390/life13051110

**Published:** 2023-04-29

**Authors:** Svetlana Kišidayová, Nikola Scholcová, Katarína Mihaliková, Zora Váradyová, Peter Pristaš, Stanislava Weisskopf, Tomáš Chrudimský, Alica Chroňáková, Miloslav Šimek, Vladimír Šustr

**Affiliations:** 1 Institute of Animal Physiology, Centre of Biosciences, Slovak Academy of Sciences, 040 00 Košice, Slovakia; 2 Institute of Soil Biology and Biogeochemistry, Biology Centre AS CR, 370 05 České Budějovice, Czech Republic; 3 Institute of Hydrobiology, Biology Centre AS CR, 370 05 České Budějovice, Czech Republic

**Keywords:** ciliates, hindgut protozoa, *Nyctotherus*, tropical millipedes, in vitro growth, enzymatic activities, polysaccharide fermentation

## Abstract

In this paper, the growth requirements, fermentation pattern, and hydrolytic enzymatic activities of anaerobic ciliates collected from the hindgut of the African tropical millipede *Archispirostreptus gigas* are described. Single-cell molecular analysis showed that ciliates from the millipede hindgut could be assigned to the *Nyctotherus velox* and a new species named *N. archispirostreptae* n. sp. The ciliate *N. velox* can grow in vitro with unspecified prokaryotic populations and various plant polysaccharides (rice starch-RS, xylan, crystalline cellulose20-CC, carboxymethylcellulose-CMC, and inulin) or without polysaccharides (NoPOS) in complex reduced medium with soluble supplements (peptone, glucose, and vitamins). Specific catalytic activity (nkat/g of protein) of α amylase of 300, xylanase of 290, carboxymethylcellulase of 190, and inulinase of 170 was present in the crude protein extract of *N. velox*. The highest in vitro dry matter digestibility was observed in RS and inulin after 96 h of fermentation. The highest methane concentration was observed in xylan and inulin substrates. The highest short-chain fatty acid concentration was observed in RS, inulin, and xylan. In contrast, the highest ammonia concentration was observed in NoPOS, CMC, and CC. The results indicate that starch is the preferred substrate of the *N. velox*. Hydrolytic enzyme activities of *N. velox* showed that the ciliates contribute to the fermentation of plant polysaccharides in the gut of millipedes.

## 1. Introduction

Ciliated protozoa often colonize the gastrointestinal tract of herbivorous animals. They are common inhabitants of the foregut of ruminants and marsupials and in the colon of many ungulates, large primates, fish, amphibians, and reptiles, as well as some birds and invertebrates. Ciliates of the family Nyctotheridae are often found in the hindgut of cockroaches and millipedes, as well as reptiles, amphibians, and fish [1]. Previous physiological studies have been devoted mainly to their energetic metabolism in specialized cellular organelles—hydrogenosomes—and their relationships with mutualist methanogenic archaea and their hosts [2,3,4,5]. To date, *Nyctotherus ovalis* from cockroaches and *Nyctotherus* species from frogs have been successfully grown in vitro [6,7,8,9]. Millipedes are litter transformers (detrivores) that feed on decomposing plant material and play an important role in nutrient cycling in soil. A stable microbial community inhabits the intestinal tract of tropical millipedes (prokaryotes and eukaryotes), and it can degrade plant polysaccharides [10,11,12]. Taylor [13] and Šustr et al. [14] hypothesized that most cellulose and hemicellulose degradation occurs in the midgut of millipedes they studied (*Orthoporus ornatus*, *Comanchelus* sp., *Archispirostreptus gigas*, and *Epibolus pulchripes*). Little is known about the fermentation of polysaccharides in the millipede hindgut. *Nyctotherus* species are considered commensal protozoa. In addition, little is known about the ability of *Nyctotherus* species and bacteria to ferment different substrates and their participation in the hindgut fermentation of their host. The study deals with some physiological aspects of *Nyctotherus* ciliates obtained from the hindgut of the tropical African millipede, *Archispirostreptus gigas*, and cultivated for a prolonged period under in vitro conditions. The growth requirements, fermentation pattern of the microbial consortium of *Nyctotherus* culture (prokaryotes and ciliates), and hydrolytic enzymatic activities of these ciliates were studied to reveal their participation in the hindgut fermentation of the host. The ciliate *Nyctotherus velox* was morphologically described because no valid description of that species has been proposed in the literature [1]. In addition, a new species of the genus *Nyctotherus* that cohabited the hindgut of A. gigas was described. The new name *N. archispirostreptae* was proposed.

## 2. Materials and Methods

### 2.1. Isolation of Ciliates

The hindgut content of African millipedes, *Archispirostreptus gigas*, was used as an inoculum for in vitro cultivation. Millipedes came from the laboratory breeding of the Institute of Soil Biology and Biogeochemistry of the Biology Centre of the ASCR in České Budějovice. We transported live millipedes to the Institute of the Animal Physiology Centre of Biosciences of the SAS in Košice. Three millipede individuals were killed by keeping them in a freezer temperature of approximately −20 °C for 5 min. The dead individuals were dissected quickly, and hindgut contents were transferred into 1 mL of 30 °C warm cultivation medium (see the Section 2.3), examined microscopically for the presence of ciliates, and pooled. The number of ciliates varied significantly in hindgut contents and was low (less than 50/animal). The inoculum (0.5 mL/tube) was divided into six cultivation tubes (Schott Glass, Germany) filled with a cultivation medium of 2 mL. The inoculated tubes were fed daily by three types of the feeding substrate: (1) wheat gluten (0.015 g/L, Sigma-Aldrich) + rice starch (0.015 g/L, BDH Chemicals) + fine-ground meadow hay (1.0 g/L), (2) wheat gluten (0.015 g/L) + 13% β sitosterol-covered rice starch (0.015 g/L), and (3) rice starch (0.015 g/L). An equal volume of fresh culture medium was added into tubes every other day up to the volume of 10 mL. Then, the contents of the cultivation tubes were divided each 3–4 days (see the Section 2.3). After 2–3 weeks, only rice starch covered with 13% β-sitosterol was used as no better growth was seen with the other tested feeding substrates. The common inhabitants of the millipede hindgut (nematodes, fungi, flagellates, and amoebas) disappeared after one month of cultivation in vitro. After that, the culture of the ciliates (with an unidentified prokaryotic microbial population) was used to study the growth requirements, fermentation parameters, and polysaccharide hydrolytic activities. Ciliates were able to grow in vitro for 1.5 years.

### 2.2. Identification of Nyctotherus Species

The species composition of hindgut ciliates was determined by molecular methods using single-cell PCR of ciliates from the hindgut content of *A. gigas*. Ten ciliates were picked out from millipedes’ hindgut contents and ten ciliates were used from the stable in vitro *Nyctotherus* culture. A single live ciliate was picked out under the microscope, washed twice in a drop of sterile cultivation mineral buffer without additives, and transferred into 50 µL of 5% Chelex-100 (BioRad, California, USA) in water. Pre-incubated proteinase K (Merck, Germany) was added to the reaction mixture to a final concentration of 20 μg/mL. After proteinase treatment (55 °C for 30 min), DNA was released from the cell by heating the sample to 98 °C for 5 min. After rapid cooling to 0 °C and centrifugation (3000× *g* for 5 min), the DNA-containing supernatant was used for PCR amplification. All isolation and manipulation steps were done under aerobic conditions [15]. Primers based on conserved regions in eukaryotic 18S rDNA genes were used in the PCR amplification (EukFor 5′-AATATGGTTGATCCTGCCAGT-3′/EukRev 5′-TGATCCTTCTGCAGGTTCACCTAC-3′) [13]. The PCR mixture contained PCR buffer with 1.5 mM MgCl2, 200 μM of dNTPs, 2.5 pmol of each primer, 0.75 U of Taq DNA polymerase (Qiagen), and 1 μL of template DNA. The PCR was performed as follows: after the initial denaturation step at 95 °C for 5 min, 35 cycles of denaturation (94 °C for 1 min), primer annealing (52 °C for 1 min), and extension (72 °C for 1 min) steps were run, followed by final extension step at 72 °C for 10 min. Quality and quantity of amplicons were checked by gel electrophoresis on 1% agarose gel in 1x TAE buffer and by measuring DNA concentration using NanoDrop^®^ ND-1000 Spectrophotometer (Thermo Fisher Scientific). PCR amplicons were purified by ExoSAP-IT reagents (Affymetrix) and bi-directionally sequenced using the 3730 Series Genetic Analyzer (Applied Biosystems). Using an iterative E-INS-I algorithm, the nucleotide sequences were aligned in MAFFT version 7 [16]. All ambiguously aligned and unaligned sites and gaps were deleted from the dataset in BioEdit [17]. The edited dataset was then used to infer phylogeny. Phylogenetic trees were constructed using Bayesian Inference (BI), Maximum Likelihood (ML), and Maximum Parsimony (MP) using MrBayes 3.2.7 [18], PhyML 3.1 [19], and PAUP* 4.0 [20], respectively. BI trees were inferred using GTR + I + Γ model with gamma distribution in six categories. The program was run for 6,000,000 generations with a sampling frequency of 100. The consensus was computed from 50,000 trees. ML trees were inferred under GTR + I + Γ model with gamma distribution in six categories. Finally, MP was performed using a heuristic search with random sequence addition and branch swapping algorithm TBR. For both ML and MP, 100 bootstrap replicates were performed, and values were used to support topologies that resulted from the BI statistically. Nucleotide sequences were deposited at NCBI. The list and description of sequences are summarized in the Supplementary Materials.

The ciliates were microscopically described, according to Albaret [1]. Before the morphological description of the ciliates, the description of the orientation of the cells in the pictures is essential. The viewer looks at the right side of the ciliate when the buccal entry (peristome) of the ciliate is on the right side. Similarly, the left side of the ciliates is assigned to the side of the cell with the peristome on the left side [1]. During light microscopy, the trophozoites are usually laid on either the left or the right side. The dorsal or ventral sides of ciliates were rarely seen. The protozoan genus and species were identified according to the size and shape of cells, macronucleus, peristome, infundibulum, contractile vacuole, cytopyge, and ciliature. Different staining procedures were used to stain intracellular amylopectin (iodine solution), nuclei (methyl-green-formalin-saline or chrome-alum-carmine), and infraciliature (pyridinated silver carbonate method) [21,22].

The pictures of ciliates were taken under bright field illumination by Moticam Pro CCD Camera (Motic Incorporation Ltd., Hong Kong) mounted on a BA400 microscope (Motic Incorporation Ltd., Hong Kong). In addition, the UV autofluorescence of methanogens in ciliates was observed with the Olympus BX51 fluorescence microscope (Olympus, Japan). Because of the presence of specific fluorescent coenzymes, methanogens can produce blue and green autofluorescence at wavelength 350 nm (coenzyme F350) and 420 nm (coenzyme F420) [23]. Specimens for scanning electron microscopy (SEM) were prepared according to Foissner [21] and observed with an electron microscope JSM-7401F JEOL (JEOL Ltd., Tokyo, Japan, acceleration voltage, 0.1–30 kV, resolution, 1 nm).

### 2.3. Cultivation of Nyctotherus In Vitro

The method of rumen ciliates cultivation was adopted. A stable culture of the ciliates with an unidentified prokaryotic population was grown anaerobically in 10 mL of the mineral medium of simplex type [24] in glass tubes with screwed caps (Schott Glass, Germany). Mineral medium composition (g/L): K_2_HPO_4_, 5.08; KH_2_PO_4_, 4.0; NaCl, 0.52; CaCl_2_, 0.036; MgSO_4_ × 7 H_2_O, 0.072; and NaHCO_3_, 6.5. The mineral medium was supplemented with (g/L): cysteine-HCl, 0.2; D-glucose, 0.4; peptone, 1.0 (Protease Peptone, Sigma-Aldrich, Inc.), 1.0 mL of trace element solution [25], and 10 mL of vitamin solution [25]. All components were of analytical grade. Before use, the medium was bubbled for 30 min with a gaseous mixture (5% H_2_ + 5% CO_2_, in nitrogen) to obtain a negative oxidation-reduction potential (ORP), which is essential for anaerobiosis (ORP, 234 ± 63 mV and pH 7.6 ± 0.2 after gassing). The complex medium was labeled as Medium A. Ciliates were fed daily with plant substrates of 0.015 g/L of the culture. The feeding substrate was resuspended in distilled water before feeding, and appropriate aliquots were pipetted into tubes. After feeding, the space above the medium was flushed out with the gaseous mixture. The culture tubes were kept at 30 °C in an incubator (Jouan, Trigon, Bratislava, Slovakia). Every 4–5th day, the cultures were divided into two halves and filled with a fresh medium of the same volume. After the establishment of the culture (about two months), we maintained the stock culture of at least 10 tubes and fed it daily with rice starch covered with 13% β-sitosterol (Sigma-Aldrich, Inc.) [26]. Initiation of the culture from cysts harvested from in vitro cultures was unsuccessful. No filamentous bacteria grew in the culture. In *Nyctotherus* culture, prokaryotes of cocci and rod morphotypes were mainly observed.

### 2.4. Experiments on the Growth Requirements of the Nyctotherus

The growth experiments lasted for three months in six replicates for each parameter. On the day of culture dilution, we collected samples from replicates by taking 1 mL of well-mixed culture and conserving with the same volume of 8% formaldehyde solution to count ciliates and bacteria.

#### 2.4.1. Experiment 1

The growth at 23, 30, 35, and 39 °C was examined. Two media and feeds were tested. Medium A was identical to the complex medium of stock culture. The soluble supplements were omitted from Medium C (peptone, glucose, trace elements, and vitamins). The rice starch (RS) and rice starch covered with 13% β-sitosterol (SRS) were used as feed substrates. Other growth conditions were the same as those of the stock culture.

#### 2.4.2. Experiment 2

The growth with different polysaccharide feeds (substrates) was investigated. The ciliate growth was examined with no particulate substrate (NoPOS), with xylan (Xylan from beech wood, Sigma-Aldrich), carboxymethyl cellulose (CMC, Sigma-Aldrich), crystalline cellulose (CC, Sigmacel 20, Sigma-Aldrich), rice starch (RS, ANALAR, Hopkins and Williams GB), and inulin (Inulin from Dahlia tubers, Sigma-Aldrich).

#### 2.4.3. Experiment 3

The growth under aerobic and anaerobic conditions was examined. Under aerobic conditions, the gas mixture and reductive agent (cysteine-HCl) were omitted. Tubes were covered only with tin lids to allow for a free escape of fermentation gas. Other conditions were the same as in the stock culture.

### 2.5. Counting of Ciliates and Total Bacteria

Trophozoites and cysts of *Nyctotherus* were counted microscopically in the aliquots of well-mixed formaldehyde-fixed samples using a mechanical counter using the method of Coleman [27]. At least four replicates were counted per sample. Samples of 12–32 per treatment were counted. The abundance of total bacteria was estimated by direct bacterial count through image analysis of pictures taken under bright field illumination of dried smears of formaldehyde-fixed samples. Two smears of known dimension and known volume per sample stained with methylene blue were prepared using the Breed method [28]. For each smear, 40 randomly selected pictures were taken at an objective magnification of 100× (Camera MoticamPro 252A mounted on the microscope Motic BA400). Images were processed and analyzed with ImageJ software according to ImageJ Software documentation [29]. Protozoa and bacteria counts were expressed per mL (in a natural logarithm, Ln) as means ± standard deviation.

### 2.6. Morphometry of Trophozoites and Cysts

The effect of the substrates on the size of *Nyctotherus* trophozoites and cysts was evaluated. The pictures were taken of the formaldehyde-fixed samples of the Nyctotherus fed by different substrates with the Moticam Pro CCD Camera (Motic Incorporation LTD., Hong Kong) mounted on the BA400 microscope (Motic Incorporation LTD., Hong Kong). The length and width of about 20 individuals of calibrated pictures were measured using ImageJ software [30].

### 2.7. Measurement of Fermentation Activity

The in vitro gas technique was used to measure the fermentation activity of the microorganism community of *Nyctotherus* culture [31]. The in vitro dry matter digestibility (IVDMD), gas volume, methane, ammonium nitrogen, short-chain fatty acids (SCFA), and the number of ciliates after 96 h of fermentations were measured in 50 mL graduated gas-tight glass syringes at 30 °C under anaerobic conditions. The aliquots of 35 mL of stock culture with six substrates (0.25 g per syringe; xylan, carboxymethylcellulose, crystalline cellulose20, rice starch, inulin, NoPOS) and four replicates per substrate were used. No gas space was left above the inoculum. After 96 h of incubation, the volume of accumulated gas was measured. The volume of produced gas was read directly from the graduated fermentation syringe. At the end of incubation, gas from each fermentation syringe was collected in a 2 mL glass syringe (for each fermentation syringe separately) and immediately analyzed for methane concentration using a gas chromatograph with FID detector (Perkin-Elmer Clarus 500; Perkin-Elmer, Inc., Shelton, CN, USA). The percentage of methane was expressed per 1 mL of gas volume. The concentration of SCFA in the fermentation medium was determined by gas chromatography using crotonic acid as the internal standard [32]. The concentration of ammonium N was determined by phenol–hypochlorite reaction [33]. The in vitro dry matter digestibility was determined from the difference in the weight of the substrate before and after incubation and the dry weight (DM) of the control group (syringes without substrates). The contents of the fermentation syringes were centrifuged at 3500× *g* for 10 min, and the residues were washed twice with distilled water, centrifuged again, and dried at 105 °C to constant weight [34]. Metabolic hydrogen recovery (HR, net amounts of “metabolic hydrogen” produced and recovered in reduced end-products formed) was calculated as the ratio between total hydrogen incorporated and produced [35,36].

### 2.8. Measurement of Hydrolytic Enzymatic Activities

#### 2.8.1. Harvesting the Ciliates

To measure enzymatic activities, the ciliates growing on rice starch were harvested in the stationary phase (after 6 days of growth and after 24 h of starvation). After 24 h starvation, the free rice starch particles were not detectable microscopically in the culture. Two-thirds of the liquid part of the culture was sucked off, and ciliates were pooled and centrifuged for 5 min at 1000 rpm (Heraeus Labofuge 400 Centrifuge, Thermo-Scientific). The bacterial population was reduced by washing five times with a sterile mineral medium without the addition of supplements. Then, the ciliates were concentrated in one polypropylene microtube (1.5 mL, Eppendorf). On average, 250,000 ± 150,000 ciliates were concentrated into one sample (one microtube) from a volume of 800–1000 mL. The concentrated ciliates were stored at −70 °C until analysis. Ciliates were gradually harvested from a culture volume of approximately 27 L.

#### 2.8.2. Preparation of Crude Protein Extracts for the Measurement of Hydrolytic Activities

Thawed samples of concentrated ciliates were diluted at a 1:5 ratio in the phosphate-citrate buffer, pH 7.2 [37], containing a protease inhibitor cocktail (Complete Mini EDTA-free protease inhibitor cocktail tablets, Roche Diagnostics, Meylan, France). Cells were disrupted by sonication on ice (10 times for 1 min per sample, 100 W, Ultrasonic Homogenizer 4710 Series, Cole-Parmer Instrument Co., Chicago, IL). Cooling of the sample for one minute was performed within the sonication intervals. Only the suspension without intact cells was used for the measurement of hydrolytic activities. The presence of intact cells was examined microscopically. After sonication, the sample was centrifuged at 6000 rpm for 10 min at 8 °C (Juany Bri Centrifuge, Thermo-Scientific). After centrifugation, the supernatant was resuspended into polypropylene mini tubes (1.5 mL, Eppendorf). Supernatants were labeled as crude protein extracts and stored in the freezer at −70 °C until analysis.

#### 2.8.3. The Measurement of Protein Concentration

The Bradford method was used to determine the protein content of the crude protein extracts [38]. The bovine serum albumin (Sigma-Aldrich, Inc.) was used as the protein standard.

#### 2.8.4. The Measurement of Hydrolytic Activities

To measure hydrolytic activities, the method of measuring the formation of reducing sugars with dinitrosalicylic acid (DNSA) was used [39]. Enzymatic activities of α-amylase, carboxymethyl-cellulase, xylanase, and inulinase were measured against respective 1% polymeric substrates (starch, CM-cellulose, 4-o-methyl-D-glucurono-D-xylan, and inulin) [40,41,42]. Enzymatic reactions were performed at 30 °C in the phosphate-citrate buffer, pH 7.2, for three hours in duplicates. Hydrolytic activities were measured for each enzyme separately in at least two assays. Enzymatic activities were expressed in SI units, katals (1 kat = 1 mol/s). Specific catalytic activities were expressed as nanokatals of reducing sugar equivalent produced per gram of protein (nkat/g).

### 2.9. Statistical Analysis

Kruskal–Wallis nonparametric analysis with Dunn’s multiple comparison tests was used to estimate the effects of growth conditions (temperature and substrates) on the counts and size of ciliates and bacteria. One-way ANOVA with Bonferroni’s Multiple Comparison Test was used to estimate differences in the parameters of fermentation activity. Treatment effects were determined to be significant at *p* < 0.05. GraphPad Prism software (GraphPad Software 5.0, Inc. San Diego, CA, USA) was used for statistical evaluations.

## 3. Results

### 3.1. Effects of Temperatures and Soluble Nutrients

**Figure 1:**
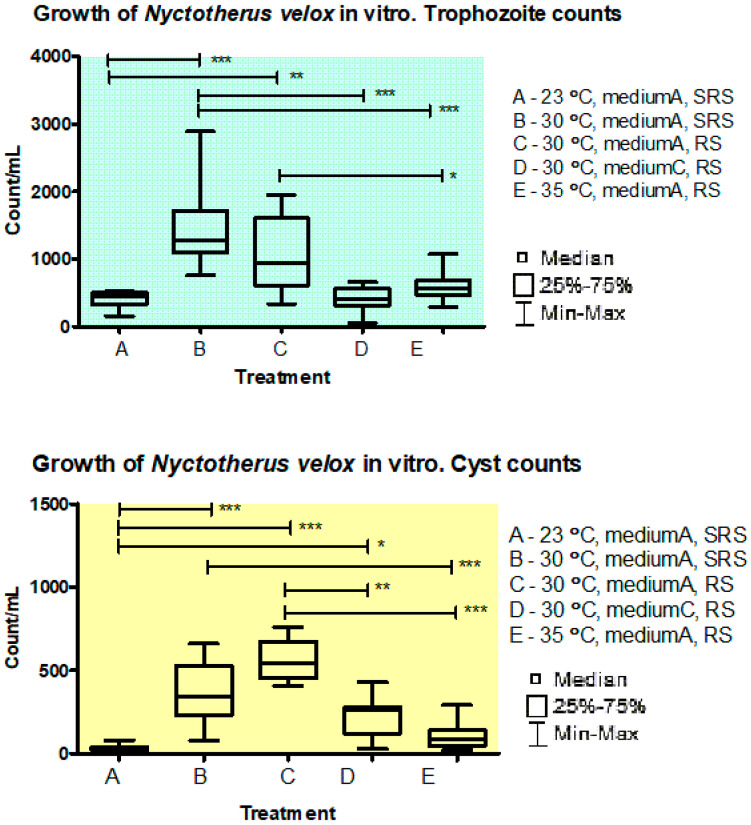
Growth of *Nyctotherus velox* in vitro. Effect of temperature, soluble nutrients, and β-sitosterol on the trophozoites and cyst counts. Medium A, complex cultivation medium. Medium C, cultivation medium without soluble nutrients (peptone, glucose, and vitamins). SRS, rice starch covered with β-sitosterol. RS, rice starch. Probability values: * *p* < 0.05, ** *p* < 0.01, *** *p* < 0.001.

The trophozoites were able to grow within a temperature range from 23 °C to 35 °C (Figure 1). At 39 °C, the trophozoites died within 24 h. The best growth was observed at 30 °C with the complex medium and β-sitosterol rice starch (or rice starch) substrate (Figure 1, 1420 ± 81/mL of trophozoites, 560 ± 36/mL of cysts, mean ± SEM). The presence of β-sitosterol on rice starch had no significant effect on the growth of trophozoites and cysts at 30 °C in comparison to rice starch. The growth also depended on the presence of soluble nutrients. Omitting soluble nutrients from the medium (peptone, glucose, and vitamins) resulted in a lower abundance of trophozoites and cysts (Figure 1, 415 ± 48/mL of trophozoites, 214 ± 34/mL of cysts, mean ± SEM).

### 3.2. Effects of Plant Polysaccharide Substrates

The effects of polysaccharide substrates on *Nyctotherus* growth are summarized in Table 1. The type of substrate influenced not only ciliates counts, but also the size of trophozoites. The counts of ciliates were also influenced by the age of the culture. The best growth was observed in the first six months of cultivation. The best growth was observed for rice starch with β-sitosterol at 30 °C (1420 ± 80/mL, Figure 1). Then, the growth decreased gradually (690 ± 240/mL on rice starch, Table 1). The Nyctotherus culture died after 1.5 years. Among the polysaccharide substrates tested, rice starch stimulated the growth of ciliates best. On the other hand, ciliates could grow without tested insoluble plant polysaccharides for an extended time (about 30 days). The tested substrates had no effects on the counts of total prokaryotes abundance except for CMC. Direct counting of bacteria revealed their decreased growth with the CMC substrate (Ln 20.685/mL, Table 1). The tested substrates also influenced trophozoite size. The smaller ciliates were observed under in vitro conditions compared to in situ. The smallest ciliates grew under the conditions in vitro with the CMC substrate (Table 1). The formation of cysts was decreased only by inulin compared to rice starch. The type of substrate did not affect the size of cysts.

### 3.3. Growth in Aerobic Conditions

The ciliates could not grow for a prolonged time under aerobic conditions. The ciliates died within 20 days. It seems that the *Nyctotherus* trophozoites can tolerate aerobic conditions for a shorter time (up to several days). In aerobic conditions, the bacteria overgrew in the culture.

### 3.4. Fermentation Patterns

The effects of substrates on the fermentation pattern of the xenic culture of the *Nyctotherus* ciliates are presented in Table 2. The highest in vitro dry matter digestibility (IVDMD) was observed after fermentation of the rice starch (92.46 g/kg DM substrate) followed by inulin (88.3), CMC (59.3), CC (50.6), and xylan (48.1). The results of IVDMD of rice starch differed significantly in comparison to CC and xylan (*p* < 0.05). The results of IVDMD of inulin differed significantly in comparison to xylan and CC (*p* < 0.05). The rice starch and inulin supported the best gas production (174 and 150 mL/g DM substrate), followed by xylan (75 mL/g DM), CMC (16 mL/g DM), and CC (4 mL/g DM). Gas production from rice starch significantly differed in comparison to xylan, CMC, and CC (*p* < 0.001). Gas production from rice starch was not significantly different in comparison to inulin. Gas production from inulin significantly differed in comparison to xylan (*p* < 0.05), CMC (*p* < 0.001), and CC (*p* < 0.001). Gas production from xylan significantly differed in comparison to CC (*p* < 0.05). The concentration of methane in fermentation gas was significantly highest for xylan (17.9%) in comparison to the other substrates (CMC, 4.11; CC, 8.77; RS, 5.29; inulin, 10.2; NoPOS, 3.85%). No differences were observed for the other substrates compared to each other. The highest concentration of ammonia was observed for CC (148.63 mg/L) followed by CMC (144), NoS (143.5), RS (32.88), inulin (30.78), and xylan (20.94). Ammonia concentrations after CMC, CC, and NoS fermentations were significantly higher in comparison to the concentrations of ammonia after xylan, RS, and inulin fermentations. The significantly highest concentrations of short-chain fatty acids (SCFA) were observed after fermentation of RS and inulin (45.5 and 40.6 mmol/L). The lowest SCFA concentrations were observed after CMC, CC, and NoS fermentations (3.5, 5.4, and 5.6 mmol/L). The acetate (from 63 to 68 mol%), propionate (from 22 to 30 mol%), and n-butyrate (from 6 to 9 mol%) were the dominant SCFA after the fermentation of xylan, RS, and inulin. On the other hand, higher proportions of acetate (from 75 to 84 mol%) were observed at the expense of propionate (from 1 to 14 mol%) and n-butyrate (from 0.5 to 1.7 mol%) after the fermentations of CMC, CC, and without substrate. Higher proportions of some minor SCFA (i-butyrate and i-valerate) were observed after the fermentations of CMC, CC, and without substrate. The highest hydrogen recovery was observed after the fermentation of xylan (80%). The lowest hydrogen recovery was observed after the fermentation of CMC (28%). No significant changes in the counts of the trophozoites after 96 h of fermentation were observed. In contrast, cyst counts were influenced by substrates. The highest count of cysts was observed in the fermentation syringes with NoPOS (263/mL) and CMC (243/mL) in comparison to RS (105/mL).

### 3.5. Enzyme Hydrolytic Activities

All examined enzyme activities were observed in the crude cell extracts of *Nyctotherus* cells (Table 3). The highest catalytic activity was observed in xylanase followed by inulinase, α-amylase, and CM-cellulase activities (µkat/L, 91.9, 87.8, 59.8, and 14.9). On the other hand, the specific catalytic activity of α-amylase was highest followed by xylanase, CM-cellulase, and inulinase (nkat/g protein, 300, 290, 190, and 170).

### 3.6. Molecular and Morphological Description of Ciliates

**Figure 2:**
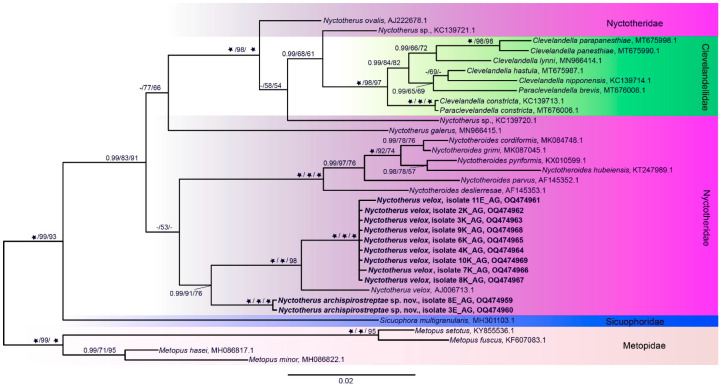
Phylogeny of the *Nyctotherus velox* and *Nyctotherus archispirostreptae* n. sp., the ciliates isolated from the hindgut content of the millipede *Archispirostreptus gigas*. Sequences obtained in this study are in the bolded text (3E, 8E, 11E, single-cell samples from the hindgut contents; 2K–10K, single-cell samples from the culture). Phylogeny of the Nyctotherus species was based on 18S rDNA gene sequences. The phylogenetic tree was constructed using Bayesian Inference (BI). The topology is further supported by Maximum Likelihood (ML) and Maximum Parsimony (MP). The first number at the nodes, followed by ML and MP bootstrap values, indicates posterior probabilities. The star indicates the maximum possible support (posterior probability 1, bootstrap 100). The presence of the dash denotes posterior probability less than 0.90 and bootstrap less than 50. The scale bar represents two substitutions per 100 nucleotide positions. The sequences are listed in the Supplementary Materials.

**Figure 3:**
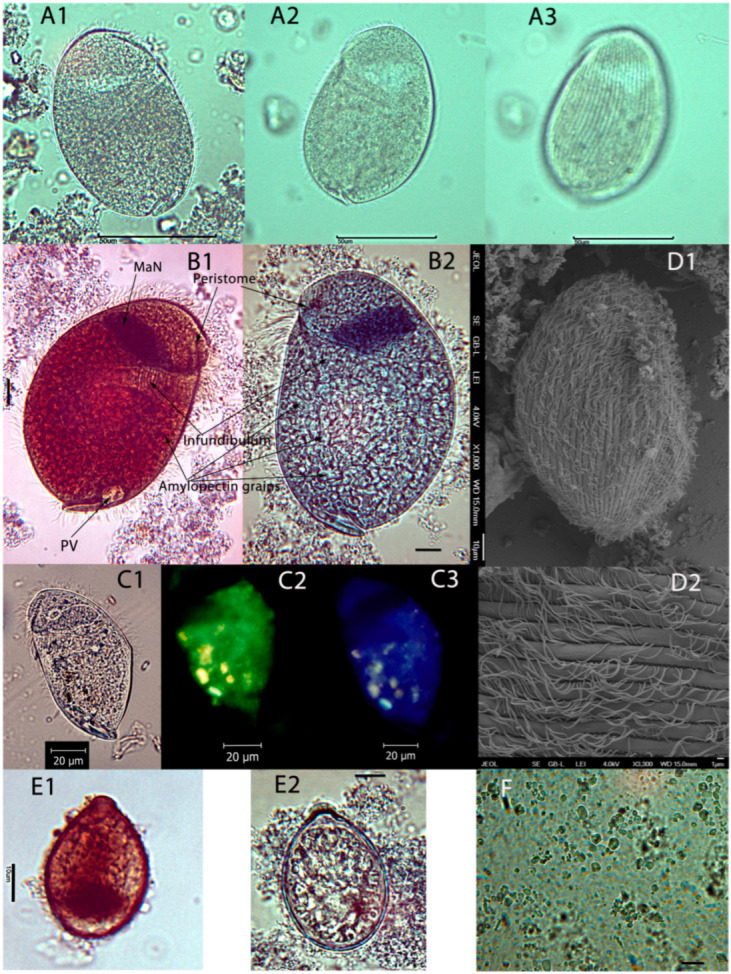
Photomicrographs of *Nyctotherus velox* from the hindgut of the millipede *Archispirostreptus gigas*. Samples were colorized by iodine (**B1**) and chrome-alum-carmine solutions (**B2**,**E2**), and the pyridinated silver carbonate method (**E1**). Scale bars are 1 µm (**D2**), 10 µm (**B1**,**B2**,**D1**,**E1**,**E2**,**F**), 20 µm (**C1**–**C3**), and 50 µm (**A1**–**A3**). Samples from hindgut contents (**A1**–**A3**); samples from the culture (**B1**,**B2**,**C1**–**C3**,**D1**,**D2**,**E1**,**E2**,**F)**; the protozoan right side (**A1**); the protozoan left side (**A2**); the striation pattern of ciliature (**A3**); MaN, the macronucleus; PV, the pulsatile vacuole; the green and blue autofluorescence of intracellular methanogens in UV light (**C2**,**C3**); the scanning electrograph of the ciliate right side (**D1)**; the scanning electrograph of the dikinetid cilia growing from the furrows (**D2**); cysts (**E1**,**E2**); intracellular amylopectin from broken ciliates fed by rice starch (**F**).

Single-cell PCR of trophozoites of the hindgut contents of *Archispirostreptus gigas* revealed the presence of at least two species (Figure 2). One sequence from hindgut contents can be assigned to the species *Nyctotherus velox* (11E). Two sequences (3E, 8E) from hindgut contents probably present species that have not been molecularly described yet. It can be assigned to the genus *Nyctotherus*. Eight sequences from the ciliates grown in vitro were assigned to the species *N. velox*. Molecular results and morphological observation pointed to only one species in the culture (Figure 3).

Taxonomic summary
Phylum Ciliophora Doflein, 1901
Subphylum Intramacronucleata Lynn, 1996
Class Armophorea Lynn, 2004
Order Clevelandellida de Puytorac and Grain, 1976
Family Nyctotheridae Amaro, 1972
Genus *Nyctotherus* Leidy, 1849

Description of *Nyctotherus velox*. In vitro growing trophozoites were pear-shaped when observed from the left and the right side (Figure 3). From either dorsal or dorsal-left (-right) sides, ciliates appeared as plum-shaped or ovoid (Figure 3). In samples from hindgut contents, the trophozoites are either oval or ovoid. The dorsal side is usually more convex than the ventral one. In some trophozoites, a large depression was observed on the ventral side under the peristome and in the plane of the infundibulum (Figure 3). The body surface was longitudinally furrowed (Figure 3D1,D2). The surface furrows labeled as creases can be described as sharp indentations when seen in cross-sections [43,44,45].

The creases were regularly spaced 2.7 µm apart (2.4–3.0 µm, SD = 0.18, n = 17). That striation pattern was also visible under the light microscope (Figure 3A3). The number of creases was estimated at 31 from the dorsal side and about 27 from the left side. Cilia as dikinetids grew from the creases (Figure 3D2) [46]. The body was uniformly (holotrichous) covered by cilia from the apical to caudal poles. The apical pole was rounded. The caudal pole was tapered from the dorsal and ventral regions (Figure 3D1). The region ventral to the anus (cytopyge) slit was prolonged into the caudal ventral lobe (Figure 3B1,B2,C1). From a dorsal point of view, the caudal ventral apex was longer, approximately 3 µm (2.3–5.8 µm, SD = 0.76, n = 28). This enlargement of the caudal ventral apex was not so pronounced in trophozoites from samples of hindgut contents. The cytopyge slit between lobes was usually narrow in trophozoites from samples of hindgut contents. The single pulsatile vacuole was situated near the ventral side of the cytopyge slit near its point of origin in the ventral caudal lobe. The in vitro grown trophozoites were filled with storage polysaccharide grains and amylopectin and colored brownish-red by iodine solution. Polysaccharide grains from the sonicated samples of the ciliates were polygonal particles (Figure 3F). It is unclear whether the grains were flat or had a polyhedral shape. The particle lengths ranged from very small (less than 1 µm) to 7 µm (mean = 3.5 µm, SD = 1.31, n = 67). The analysis of the particle area distribution revealed that the particles of 1.2 µm^2^ prevailed (count 32 of 67). The polygonal shape of the grains was also visible in the intact ciliates from in vitro culture and hindgut samples. In hindgut samples, trophozoites contained far fewer storage grains, and they were of smaller size. In vitro, the mass of storage grains obscured the details of the other structures in the cell endoplasm except for the macronucleus, infundibulum, and pulsatile vacuole. Storage polysaccharide grains were also visible in cysts. Peristome started near the apical pole, extended over one-third of the cell, and divided that part into left and right lips, which were covered inside by the tufts of cilia (membranelles). In one-third of the cell, the peristome entered the cell and continued as an infundibulum at the angle of 93–116° from the longitudinal cell axis (mean = 107°, SD = 5.7, n = 14; measured either on the left or right cell side). The infundibulum was a straight funnel-shaped tube, 27 µm long on average (21–36 µm, SD = 3.7, n = 21), and directed to the dorsal surface. In the entry into the cell, the infundibulum was 7 µm width on average (4–10 µm, SD = 1.6, n = 21). The inner surface of the infundibulum was regularly transversely striated. It is probable, that tufts of cilia (membranelles) can grow from the infundibulum creases. The cilia or tufts of cilia were not visible in the infundibulum under light microscopy. The cytopharynx and cytostome were not visible. The mean trophozoites area was 3270 µm^2^ (1770–4283 µm^2^, median = 3235 µm^2^, SD = 775.8, n = 15), mean length was 79 µm (56–91 µm, SD = 10.2, n = 15), mean dorsoventral width was 53 µm (41–63 µm, SD = 6.4, n = 15), and mean L/W ratio was 1.5 (1.3–1.6, SD = 0.08, n = 15) of *N. velox* from hindgut contents (in vivo). Table 1 presents the mean length and dorsoventral width of trophozoites and cysts of cultures fed different substrates in vitro. Trophozoites’ mean lengths (L) varied from 51 µm to 75 µm, the dorsoventral width (W) varied from 36 µm to 50 µm, and the L/W ratio varied from 1.5 to 1.7. The length of the left-right axis varied from 37 µm to 51 µm (mean = 44 µm, SD = 6.3, n = 4). The different feeding conditions produced trophozoites of different sizes. The ciliates were smaller in cultures with no particulate polysaccharide substrate or substrate with too large feeding particles (CMC). The macronucleus (MaN) was situated almost horizontally in the karyophore in the anterior part of the cell just above the infundibulum. The shape was often lenticular, sometimes an inverted pyramid or trapezoid. MaN was a relatively large organelle: mean MaN area was 300 µm^2^ (150–470 µm^2^, SD = 94.4, n = 22), mean length was 30 µm (21–40 um, SD = 5.5, n = 22), mean width was 14 µm (10–18 µm, SD = 2.4, n = 22), and mean L/W ratio was 2.3 (1.8–2.7, SD = 0.28, n = 22). It was hard to distinguish the micronucleus from the mass of the macronucleus. The blue and green autofluorescence of intracellular methanogens was observed (Figure 3C2,C3), which points to the presence of both coenzymes (F350 and F420) in the intracellular methanogenic archaea.

Description of cysts: The cysts were oval with a thick wall and a single small round protrusion (Figure 3E1,E2). When viewed from the protrusion side, the cysts were round. The oval macronucleus usually lay in the cyst’s lower half near the center. The cysts contained storage polysaccharide grains. The cyst size was not influenced by the tested in vitro conditions (Table 1). The mean area of cysts was 707 µm^2^ (497–998 µm^2^, SD = 102.2, n = 83), the mean length was 35 µm (28–41 µm, SD = 2.5, n = 83), the mean width was 28 µm (23–34 µm, SD = 2.2, n = 83), and mean L/W ratio was 1.2 (1–1.4, SD = 0.05, n = 83).

Type locality: Hindgut of the millipede *Archispirostreptus gigas*.
Type host: *Archispirostreptus gigas* Peters, 1868
Country of the host origin: Tanzania
Gene sequences. The nuclear 18S rDNA gene sequences were deposited in GenBank. Accession numbers are listed in the Supplementary Materials.
Family Nyctotheridae Amaro, 1972
Genus *Nyctotherus* Leidy, 1849

**Figure 4:**
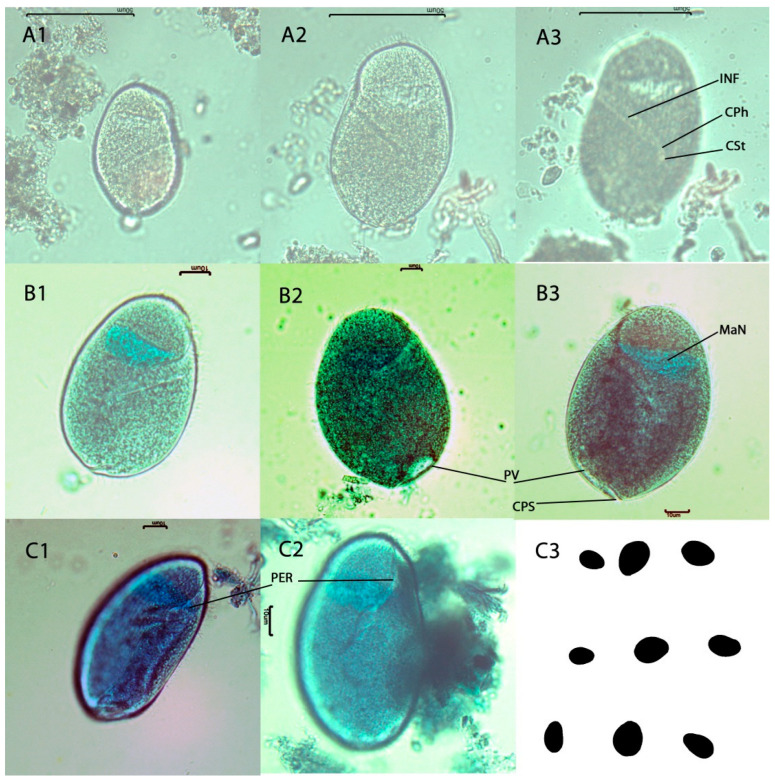
Photomicrographs of *Nyctotherus archispirostreptae* n. sp. from the hindgut content of the millipede *Archispirostreptus gigas*. The samples (**B1**–**B3**,**C1**,**C2**) were colorized by methyl-green formalin solution. Scale bars are 10 µm (**B1**–**B3**,**C1**,**C2**) and 50 µm (**A1**–**A3**). The protozoan right side (**A1**,**B1**,**B2**); the protozoan left side (**A2**,**A3**,**B3**); the striation pattern of ciliature and the infundibulum ending into cytostome (**A3**); view from the ventral-right side (**C1**,**C2**); the image segmentation illustrates the variability in ciliate size and shapes (**C3**). Inf, the infundibulum; CPh, the cytopharynx; CSt, the cytostome; MaN, the macronucleus; PV, the pulsatile vacuole; CPs, the cytopyge slit, PER, the peristome.

Description of *Nyctotherus archispirostreptae* n. sp. The pooled hindgut contents of three *A. gigas* were a source of samples as the number of ciliates was low. The trophozoites were very similar to the *N. velox*. The trophozoites were ovoid (Figure 4C3). The dorsal side was slightly more convex than the ventral one. A slight depression occurred on the ventral side, in the place of the entry of the peristome to the cell, in one-third of the cell. The body surface was longitudinally furrowed (Figure 4A3). The body was uniformly (holotrichous) covered by cilia from the apical to caudal poles. Apical and caudal poles were usually rounded. Some trophozoites had the caudal lobes pointed gently (Figure 4B2,B3). The lower part of the cell was divided by a cytopyge slit into the dorsal and ventral lobes, with a longer cutout on the left side (Figure 4B3). The cytopyge opened into the space (the slit) between these lobes. Usually, the slit was narrow. The caudal lobes had similar sizes from the left side. The pulsatile vacuole was located near the cytopyge on the ventral side of the trophozoite (Figure 4B2). The storage amylopectin grains were visible in the cells. Morphometric data are summarized in the Table 4. The mean length of trophozoites was 78 µm (61–91 µm, SD = 10 µm, n = 9), the mean width was 54 µm (42–70 µm, SD = 10.5 µm, n = 9), the mean L/W ratio was 1.5 (1.2–1.7, SD = 0.14, n = 9), and mean trophozoites area was 3274 µm^2^ (1952–4588 µm^2^, SD = 971.2, n = 9). Peristome started near an apical pole, extended over one-third of the cell, and divided that part of the cell into left and right lips (Figure 4A1,A2,B1–B3,C1,C2). The peristome was covered by membranelles. In one-third of the cell, the peristome entered the cell and continued as an infundibulum at the angle 121–126° from the longitudinal cell axis (mean = 125°, SD = 2.2, n = 4). The infundibulum was a funnel-shaped tube directed to the dorsal surface (Figure 4A1,A2,B1). Its mean length was 28 µm (21–33 µm, SD = 3.9 µm, n = 5). The inner surface of the infundibulum was regularly transversely striated. The cytopharynx and cytostome were usually not visible. The peristome narrowed into a short cytopharynx, which ended in a small, almost spherical cytostome (Figure 4A3). The macronucleus (MaN) was situated almost horizontally in the karyophore in the anterior part of the cell above the infundibulum. The shape was often lenticular, sometimes an inverted pyramid. The micronucleus was hard to distinguish from the mass of the macronucleus.

Type locality: Hindgut of the millipede *Archispirostreptus gigas*.
Type host: *Archispirostreptus gigas* Peters, 1868
Country of the host origin: Tanzania
Gene sequences: The nuclear 18S rDNA gene sequences were deposited in GenBank. Accession numbers are listed in the Supplementary Materials.
Etymology: The species name is derived from the generic name of the host.

## 4. Discussion

To date, *Nyctotherus* species of frogs and cockroaches have been cultivated, but no studies on axenic cultures have been carried out. Generally, it can be assumed that all work on the cultivation of the *Nyctotherus* species has used xenic cultures, which means that various microbes, mainly prokaryotes, were present in *Nyctotherus* cultures. Aragao [47] kept *N. cordiformis* from the frog *Leptodactyllus ocellatus* in Petri dishes with 0.5% egg white in 0.85% NaCl. He obtained a multiplication of the Nyctotheri usually in three to four days; they lived for up to a fortnight, and then either encysted or simply died. Additional feeding of the ciliates with frog blood only resulted in a stronger overgrowing of bacteria. Pinto [48] kept *N. cordiformis* alive for short time, in a physiological solution of diluted feces of the host (*Bufo marinus*) at room temperature, for up to 30 days, without multiplication or conjugation of the ciliates. Nelson [7,49] kept *N. cordiformis* from the rectum of *Rana pipiens* in an extract from rectum content in Ringer’s solution and a supplementary feeding with rice starch. The ciliates lived up to 180 days, reaching an abundance of 550 ciliates per test tube. The addition of alcoholic extracts of frog tissues (liver and stomach) extended the duration of the culture up to 455 days and increased the number of ciliates to 2200 per test tube. Lucas [50] maintained *N. ovalis* from *Periplaneta americana* for up to 19 days at 28 to 30 °C in a serum-saline medium and starch supplement. Balch [6] kept *N. ovalis* from *Blatella germanica* in 0.5% salt solution with non-inactivated rabbit serum for 40 days at room temperature. Chen [9] maintained *N. ovalis* from *Periplaneta orientalis* for more than 3 months in the albumin-serum medium at 28–30 °C. Kudo [51] maintained *N. ovalis* in Ringer´s solution with albumin supplement for several weeks at room temperature and pH 7–7.8 in Petri dishes. Lom [8] maintained *N. cordiformis* and *N. hylae* from five species of frogs, and *N. ovalis* from cockroaches (*Periplaneta americana*) and European mole crickets (*Gryllotalpa gryllotalpa*) in test tubes. He tested four media. Ciliates grew best in the Ringer´s solution (supplemented with pig caecum content rich in bacteria) at laboratory temperature and aerobic conditions. Ciliates were fed rice starch. N. cordiformis survived up to 212 days at a density of about 210/mL. *N. hylae* survived up to 168 days at a density of 100/mL. *N. ovalis* survived up to 150 days at a density of 140/mL. Recently, Suzuki et al. [52] succeeded in culturing the *N. teleacus* from the feces of a tortoise (*Astrochelys radiata*). *N. teleacus* grew in BR medium supplemented with a 4% bovine serum. No further details were provided.

In our study, the best growth of *N. velox* was observed at 30 °C in the buffered complex medium under anaerobic conditions and rice starch feeding. The ability of *N. velox* to grow in the range of temperatures of 20 35 °C agreed with other studies [7,8,48,51,53]. We and other workers have observed the best growth at 30 °C [9,50]. Soluble supplements were useful as growth promoters in our experiments and in other works too. In our opinion, these soluble supplements may serve as nutrient sources also for the accompanying prokaryotes. It is probable that engulfed bacteria are the main source of proteins for the ciliates. Similar results were also observed in other studies [6,8,50,51]. The suitability of rice starch has been confirmed in most studies referred to above. In addition, we observed relatively good growth on other plant polysaccharides (inulin, xylan, CMC, and CC). The stable density of prokaryotes was observed in experiments with different polysaccharide substrates except for CMC. CMC has very large particles that are likely difficult to digest by both ciliates and prokaryotes, resulting in lower counts of total prokaryotes. In addition, *N. velox* could grow without insoluble polysaccharide substrates for at least 30 days. It seems that the prokaryotes in culture could be a good source of nutrients for the growth of the ciliates if suitable soluble nutrients are present. It can therefore be concluded that the plant polysaccharides tested are not essential for the growth of the ciliates. Nevertheless, it can be recommended the addition of rice starch to support the stable growth of the ciliates over an extended period in vitro. It can be assumed that rice starch may stimulate the growth of ciliates as well as some populations of accompanying prokaryotes. The absence of soluble nutrients (vitamins, glucose, and peptone) resulted in low counts of trophozoites and cysts (Figure 1). Rice starch did not replace the effect of soluble nutrients. It cannot be excluded that these soluble supplements are used directly by ciliates. Further experiments are needed to determine which soluble nutrients are the best growth promoters. In addition, ciliates can probably metabolize granules of their intracellular storage polysaccharide under starving conditions.

Trophozoites fed on rice starch had plenty of granules, so their shape was pear-shaped. In contrast, trophozoites from hindgut samples had far fewer granules and were more plum-shaped. The occurrence of intracellular storage polysaccharide particles was repeatedly observed in many *Nyctotherus* species [1,8,54,55,56]. In older literature, the name paraglycogen was used. In the presented study, the reddish-brown color of *Nyctotherus* intracellular grains stained by iodine solution indicated that the grains are amylopectin or glycogen. Further research may clarify the nature of these polysaccharide grains [57,58,59]. Lom [54] described the storage polysaccharide particles of several *Nyctotherus* species from the hindgut of frogs and cockroaches as round or oval flat granules, sometimes concave or lenticular. These amylopectin-like granules ranged from very small up to 4 µm. In polarized light, the grains had a regular birefringence. In the study, amylopectin-like granules of *N. velox* were similar in size with the prevalence of the small particles (with an area of about 1 µm^2^). We observed a prevalence of polygonal-shaped grains. It is not clear whether the observed particles are products of the ciliates’ metabolism or whether they are only engulfed rice starch particles. Rice starch particles have a similar size distribution (2–7 µm) and similar polyhedral shape [60,61]. Scanning electron microscopy may reveal more details about the shape of the amylopectin-like granules of the *Nyctotherus* species.

The most controversial feature is the requirement for anaerobic conditions. In previous works, Nyctotheridae ciliates were kept in vitro without emphasis on anaerobic conditions. In previous studies, growth in Petri dishes (open system) usually lasted only for a short time. Growth in test tubes was more successful. No previous works specified whether the test tubes were closed. In laboratory practice in the past, either cotton plugs and tin lids or rubber stoppers were commonly used in these experiments. It can be inferred that the accompanying facultative anaerobes could generate low oxygen conditions in the test tube cultures. The presented experiments confirmed that aerobic conditions (test tubes with non-reduced medium and covered only with tin lids) gradually led to the death of protozoa. Recently, anoxic conditions in the hindgut of millipedes were described in a study by Horváthová [62]. In addition, most of the trophozoites preferred to swim on the bottom of test tubes, which is another sign of low oxygen preferences. Swimming of *Nyctotherus* species at the bottom of test tubes was also observed in the study by Lom [8]. These results point to the facultative anaerobic nature of *Nyctotherus* species. Other intestinal ciliates, rumen ciliates, are also facultative anaerobes and can tolerate low oxygen levels for a short period [63,64,65]. In contrast, aerobic ciliates (e.g., *Paramecium*, *Euplotes*, and *Tetrahymena*) usually swim near the air–liquid medium interface.

Cultivation conditions also influenced the size of *N. velox* trophozoites. Generally, we observed smaller trophozoites under in vitro conditions in comparison to hindgut samples. The size of cysts was not influenced by cultivation conditions. Similar effects were observed in experiments on other *Nyctotherus* species [8].

To date, no study has investigated the fermentation pattern of *Nyctotherus* species fed different polysaccharide substrates in vitro. Microbes in anaerobic ecosystems compete for metabolic hydrogen in three major ways: methanogenesis, reductive acetogenesis, and non-assimilatory sulfate reduction. In a hindgut environment, reductive acetogenesis and methanogenesis can occur simultaneously [66]. The comparisons of calculated hydrogen recovery (HR, net amounts of “metabolic hydrogen” produced and recovered in reduced end-products formed) revealed much lower HR in the hindgut environments compared to the rumen, especially when no ciliates were present in the hindgut [66]. The fermentation results showed that the microbial community in the hindgut of *A. gigas* is well-adapted to the feeding behavior of the host [11], and it can ferment different storage and fiber polysaccharides. In the study, the fermentation of different polysaccharide substrates revealed a methanogenic fermentation pattern of the *N. velox* microbial community, as did rumen ciliates, horse hindgut ciliates, and chimpanzee hindgut ciliates [67,68,69,70]. Several studies have focused on methanogenesis in the hindgut of millipedes and cockroaches [2,62,71,72]. Methanogenesis can be greatly enhanced when ciliates are present in the hindgut of tropical millipedes and cockroaches [2,62]. Methanogenic archaea use molecular hydrogen produced by anaerobic mitochondrion or hydrogenosomes of anaerobic eukaryotic microorganisms [5,73,74]. The association of anaerobic ciliates with methanogens is a well-described feature not only in *Nyctotherus* species, but also in rumen ciliates, hindgut ciliates of great apes, equine hindgut ciliates, and free-living anaerobic ciliates [75,76,77,78,79]. The relatively low HR (except for xylan) indicates the presence of additional metabolic hydrogen sinks, probably through reductive acetogenesis in the fermentation experiments. Taylor [13] and Šustr et al. [14] hypothesized that most cellulose and hemicellulose degradation occurs in the midgut of millipedes they studied (*Orthoporus ornatus*, *Comanchelus* sp., *Archispirostreptus gigas*, and *Epibolus pulchripes*). The fermentation experiment showed a remarkable proportion of cellulose and hemicellulose (xylan) degradation in the hindgut. In addition, we found activities against CMC and CC in the crude protein preparation of *Nyctotherus* cells. Enzymatic activities against cellulose were found in the gut content of several millipedes [13,14,80]. The storage polysaccharides (inulin and rice starch) were the highest digestible substrates. Taylor [13] suggested that storage polysaccharides are fermented primarily in the hindgut. We observed amylolytic activity in *Nyctotherus* crude cell extract. We hypothesize that *Nyctotherus* could contribute to starch fermentation in the hindgut. The amylolytic activities of other members of the hindgut microbiome need to be studied to compare their contributions. Amylase activities were also reported in ciliates of other anaerobic gut environments: rumen ciliates, horse hindgut ciliates, and great ape hindgut ciliates *Troglodytella abrassarti* [24,67,81,82]. Inulins are plant storage fructan polysaccharides typically found in roots or rhizomes. We hypothesized that the diet of millipedes might contain this polysaccharide, and their hindgut microbiome might ferment it. Laboratory observations reveal that *A. gigas* prefer fresh or decaying vegetables [11]. So far, no study has dealt with the inulinase activity in the hindgut contents of millipedes. The high IVDMD of inulin was comparable to that of rice starch. Enzymatic assay confirmed the presence of inulinase hydrolytic activity in *Nyctotherus* crude cell extracts. The results point to the adaptation of the hindgut microbiome of millipedes to the fermentation of inulins. Inulinase activity was also found in zebra hindgut ciliates and bacteria as well as in a bacterial fraction of chimpanzee feces [67,81].

In terms of fermentation gas volume, the concentration of short volatile fatty acids (SCFA), and ammonia, the results indicate two fermentation patterns according to the substrates used. Fermentation without substrates or with cellulosic substrates resulted in high ammonia concentration at the expense of SCFA and fermentation gas. This suggests that the microbial consortium preferentially fermented nitrogenous sources. Since *Nyctotherus* can survive in vitro for quite a long time without polysaccharides, it can be assumed that the high ammonia concentration results from the digestion of ingested bacteria and soluble nitrogen sources (peptone). In addition, the ciliates may also metabolize their intracellular amylopectin stores, as methanogenesis at non-substrates conditions was similar to those of rice starch fermentation. It cannot be excluded that this fermentation mode may occur in the hindgut of the host as an alternative mode of fermentation when the host is poorly nourished [13]. Gonzales et al. [83] observed higher ammonia concentrations and lower millipedes’ weight when lignin-rich leaf litter was present in the experimental chambers. Fermentation of CMC substrate (or without any polysaccharide substrate) led to a higher proportion of acetate at the expense of propionate and n-butyrate proportions, accompanied by lower methanogenesis and hydrogen recovery. We can speculate that fermentation could have shifted to reductive acetogenesis [66,84]. Xylan is a major component of plant hemicelluloses and can constitute up to 30% of plant tissues. In the study, the fermentation of xylan produced the highest concentration of methane in the fermentation gas and the highest hydrogen recovery, similar to the level of HR in the rumen [66]. It seems that the xylan strongly stimulated the association of the xylanolytic microbial consortium with methanogenic archaea. The observed relatively high xylanolytic activities in crude extracts of *N. velox* were comparable to amylolytic activities. In the study by Šustr et al. [14], xylanolytic activity was not observed in the midgut and the hindgut of *A. gigas* and *E. pulchripes*. These authors assume this was due to the methods used to measure this activity. On the other hand, Nunez and Crawford [80] found xylanolytic activities in the hindgut contents of the dessert millipede *Orthoporus ornatus*. The xylanolytic activity was also found in rumen ciliates, horse hindgut ciliates, and great ape hindgut ciliates *T. abrassarti* [24,67,81,82]. Observed fermentation patterns and enzymatic activities point to the excellent adaptation of *N. velox* to the diverse feeding behavior of *A. gigas*. Recently, metatranscriptomic holobiont analysis of the millipede *Telodeinopus aoutii* revealed the expression of more than 50% of carbohydrate-active enzymes in the hindgut microbiome [85].

Short-chain fatty acids (SCFA) are an important source of energy for ruminants and serve as a supplementary energy source in the hindgut tissues of monogastric animals. The importance of SCFA in the metabolism of millipedes has not been elucidated so far. The millipedes can benefit from the gut microbiota if millipedes can absorb the products of microbial fermentation from the hindgut, as shown previously in termites and cockroaches. The utilization and the active transport of volatile fatty acids across the hindgut of the cockroach (*Panesthia cribrata*) and the termite (*Mastotermes darwiniensis*) have been described [86]. Even though the structure of the hindgut of millipedes differs from that of cockroaches and termites, it cannot be excluded that millipedes use products of microbial fermentation, for example, SCFA.

The genus *Nyctotherus* and the species *N. velox* (from julid millipede) and *N. ovalis* (from *Blatta orientalis*) were first introduced and shortly described by Leidy [87,88]. The descriptions were considered insufficient, and *N. ovalis* was redescribed in detail by Albaret [1]. To date, no detailed description of *N. velox* has been carried out. Leidy described the *N. velox* as a protozoon with a white, translucent, and ovate body; anteriorly obtusely rounded; and posteriorly angular. The anterior areola was faintly granular and trapezoidal, with bulging sides. Interiorly furnished with several minute vacuoles, and usually one large and globular vacuole situated just at the end of the posterior fissure. Antero-inferiorly and middle line, the body is furnished with a semicircle of large vibrillae, anterior to which is a large, granular areola, and posteriorly, there is a short fissure passing inwards and downwards [87]. The work of Savin dealt with the other description of *N. velox* from the millipede *Spirobolus marginatus* [89]. Based on the presented drawings, we believe that there were about two species in the hindgut of *S. marginatus*. Due to the remarkable similarity, the author described them as one species. Differences are observable in the course of the infundibulum and the shape of the lower part of the ciliates. Nevertheless, Savin’s observations were very similar to our observations. Albaret [1] distinguished the genera of the Nyctotheridae family (*Pronyctotherus*, *Nyctotherus*, *Metanyctotherus*, and *Nyctotheroides*) also by the presence of kinetal suture systems (somatic kineties forming a variety of sutures or complex secant systems on the ciliate surface). According to Albaret [1], the *Nyctotherus* genus has one apical suture on the right side. Unfortunately, we were unable to identify these structures in our isolates. In the work, molecular analyses showed two types of ciliates in *A. gigas.* Molecularly, the name *N. velox* was assigned to an isolate from an unspecified julid millipede [90]. Unfortunately, no images of the isolate were presented in that work. The isolate from millipede and in vitro culture, molecularly identified as *N. velox,* resembles the drawings presented by Leidy [87]. The ciliate measurements made by Leidy appear to be in error and cannot be compared with ours. The typical features of *N. velox* are the position and shape of the macronucleus, the course of the peristome and the infundibulum, and the shape of the lower part of the cell around the anus slit. Microscopic comparison between the two species cohabited in the hindgut of *A. gigas* revealed differences in the course of the infundibulum and the shape of the lower part of the ciliate cells. Morphometry analysis revealed very similar cell and macronucleus size distributions. Few *Nyctotherus* species have been subjected to molecular analysis. The molecular data revealed a probable common origin of the *N. archispirostreptae* n. sp. and *N. velox*. The samples of *N. archispirostreptae* n. sp. form a distinct group from those of *N. velox*. They are paraphyletic to the species of amphibians (*Nyctotheroides*) and cockroaches (*Nyctotherus ovalis* and species of the Clevelandellidae family.). *Nyctotherus ovalis* and Clevelandellidae species from cockroaches formed a separated clade. When more *Nyctotherus* isolates from the millipedes are sequenced, it could determine whether millipede isolates form a clade distinct from cockroaches [91]. Our data and other studies showed that species of *Nyctotherus* and *Nyctotheroides* are paraphyletic [52,91,92,93]. Molecular analyses of the members of the order Clevelandellida have shown that *N. velox* has an unstable relationship with other species assigned to the genus *Nyctotherus* [90,91,92,93,94,95]. The order Clevelandellida is monophyletic [91,92,95], and the orders Armophorida and Clevelandellida are sister taxons [90,91,96]. To reveal taxonomic differences of the Nyctotheridae species, more detailed morphological studies (TEM, SEM, and silver impregnations) of oral apparatus and somatic ciliature are essential.

## 5. Conclusions

The presented study was the first step to understanding the complex relationships in the hindgut microbiome of millipedes. The simplified community under in vitro conditions can somewhat simulate these conditions. The study demonstrated that *N. velox* is a facultative anaerobic ciliate involved in the fermentation of plant polysaccharides in the hindgut of its millipede hosts. The observed methanogenic fermentation patterns and hydrolytic enzymatic activities point to the excellent adaptation of *N. velox* to the diverse feeding behavior of *A. gigas*. The success in the long-term growth of *N. velox* in vitro can promote further studies on the physiology of members of the family Nyctotheridae and their role in the life of their hosts. The axenic ciliate cultures are essential to study the relationships between *Nyctotherus* and specific members of the millipede hindgut environment. The study of enzymatic activities of other members of the hindgut microbiome will contribute to the knowledge of the processes in hindgut fermentation. Some issues related to these findings—such as the contribution of hindgut ciliates to millipede digestion and nutrition and, consequently, the mutualistic or commensal nature of their relationship, the necessity of ciliates for hindgut methanogenesis, the biological interpretation of differences in ciliate abundance between individual millipedes, or coevolution between ciliates and millipedes—require further study. More detailed morphological studies (TEM, SEM, and silver impregnations of oral apparatus and somatic ciliature) may find new features to differentiate the members of the Nyctotheridae family better. In addition, it is essential to study more Nyctotheridae members molecularly to reveal their phylogeny.

## Figures and Tables

**Table 1:** The effects of substrates on the number and size of the ciliate *Nyctotherus velox* and bacteria counts grown in the xenic culture established from the hindgut contents of the African millipedes *Archispirostreptus gigas*.

	Rice Starch	CMC	Celulose20	Xylan	Inulin	In Situ	NoPOS
Trophozoites							
Number, n/mL	690 ± 240 *	490 ± 160	500 ± 300	360 ± 140	390 ± 280	ND	450 ± 70
Length, µm	68 ± 9.2 *	51 ± 5.1 ***	59 ± 9.4 ***	62 ± 7.1 ***	61 ± 12.9 ***	75 ± 9.2	55 ± 6.0 ***
Width, µm	43 ± 7.9 **	33 ± 4.0 ***	42 ± 9.0 **	40 ± 5.4 ***	38 ± 12.0 ***	50 ± 8.1	36 ± 4.4 ***
L/W	1.6 ± 0.2	1.5 ± 0.2	1.5 ± 0.2	1.5 ± 0.9	1.7 ± 0.2	1.5 ± 0.2	1.6 ± 0.2
Cysts							
Number, n/mL	160 ± 100	200 ± 150	130 ± 100	140 ± 90	60 ± 40 *	ND	290 ± 90
Length, µm	30 ± 1.9	27 ± 3.9	30 ± 3.0	28 ± 1.8	33 ± 3.9	-	31 ± 0
Width, µm	24 ± 1.6	21 ± 1.2	24 ± 2.1	22 ± 2.0	24 ± 2.0	-	23 ± 0
L/W	1.3 ± 0	1.3 ± 0.1	1.2 ± 0	1.3 ± 0.1	1.4 ± 0.1	-	1.4 ± 0
Bacteria (n/mL, Ln)	22.175 ± 0.2	20.685 ± 0.5 *	22.173 ± 0.5	21.273 ± 0.3	22.387 ± 0.3	-	-

CMC, carboxymethylcellulose; NoPOS, no polysaccharides; ND, not determined; Values are means ± standard deviation; L/W, length to width ratio; * *p* < 0.05; ** *p* < 0.01; *** *p* < 0.001.

**Table 2:** The effects of substrates on the fermentation pattern of the xenic culture of the ciliate *Nyctotherus velox* established from the hindgut contents of the African millipede *Archispirostreptus gigas*.

Item		Xylan	CMC	CC	Rice Starch	Inulin	NoPOS	SE	*p*-Value
IVDMD	g/kg DMsubst	48.10 ^c,d^	59.27	50.59 ^a,b^	92.46 ^b,d^	88.30 ^a,c^	-	5.211	0.0019
Gas	mL/g DMsubst	75 ^a,d,g^	16 ^b,e^	4 ^c,f,g^	174 ^a,b,c^	150 ^d,e,f^	-	16.156	0.0001
Methane	% (vol/vol)	17.89 ^a,b,c,d,e^	4.11 ^a^	8.77 ^b^	5.29 ^c^	10.19 ^d^	3.85 ^e^	1.182	0.0001
Ammonia	mg/L	20.94 ^a,b,c^	144.00 ^a,d,g^	148.63 ^b,e,h^	32.88 ^d,e,f^	30.78 ^g,h,i^	143.50 ^c,f,i^	12.627	0.0001
SCFA	mmol/L	22.89 ^a^	3.47	5.43	45.45 ^b^	40.63 ^c^	5.60 ^a,b,c^	3.581	0.0001
Acetate	mol%	62.69 ^a^	83.89	74.78 ^b^	65.72 ^c^	67.91 ^d^	82.36 ^a,b,c,d^	1.719	0.0001
Propionate	mol%	29.72 ^a^	1.38	14.14 ^b^	21.99 ^c^	23.39 ^d^	3.50 ^a,b,c,d^	2.220	0.0001
nButyrate	mol%	6.13 ^a^	0.52	1.72	9.09 ^b^	5.83 ^c^	1.51 ^a,b,c^	0.482	0.0001
iButyrate	mol%	0.62 ^a^	5.50	3.92	0.23 ^b^	0.40 ^c^	5.22 ^a,b,c^	0.649	0.0001
nValerate	mol%	0.52 ^a^	3.27 ^b^	2.22	2.67	2.00	2.10 ^a,b^	0.185	0.0001
iValerate	mol%	0.33 ^a^	5.36	3.16 ^b^	0.28 ^c^	0.47 ^d^	5.26 ^a,b,c,d^	0.474	0.0001
2H-recovery	%	80.13 ^a^	28.23	47.91 ^b^	48.02 ^c^	53.93 ^d^	30.16 ^a,b,c,d^	3.787	0.0001
Trophozoites number, T	n/mL	608	823	650	115	800	558	70.985	0.0708
Cysts number, C	n/mL	108	243 ^b^	160	105 ^a,b^	175	263 ^a^	14.674	0.0035
Cyst proportion	%	12.05	22.75 ^b^	19.67	63.42 ^a,b^	29.98	36.06 ^a^	5.24	0.0035

Values are arithmetic means. SE, standard error; CMC, carboxymethylcellulose; CC, crystalline cellulose20; NoPOS, no polysaccharides; IVDMD, in vitro dry matter digestibility; SCFA, short-chain fatty acids; ^a–i^, values within a row with an identical superscript letter are significantly different (*p ˂* 0.05).

**Table 3:** Enzyme hydrolytic activities of the ciliate *Nyctotherus velox* grown in vitro and fed with rice starch.

Enzyme	Catalytic Activity		Specific Catalytic Activity	
	nkat/L ± SD	n	nkat/g Protein ± SD	n
Amylase	59 830 ± 71 490	2	300 ± 120	2
CM-cellulase	14 900 ± 21 830	6	190 ± 110	6
Xylanase	91 930 ± 72 440	4	290 ± 100	4
Inulinase	87 830 ± 110 300	2	170 ± 220	2

Values are arithmetic means ± standard deviation.

**Table 4:** Morphometric data of *Nyctotherus archispirostreptae* n. sp.

	Ciliate Area (µm^2^)	Ciliate Lenght (L) (µm)	Ciliate Width (W) (µm)	L/W	MaN Lenght (µm)	MaN Width (µm)	Infund. Angle (°)	Infund. Lenght (µm)
Arith. mean	3270	78	54	1.5	30	10	125	28
SD	970	10	11	0.14	4.5	2.8	2.2	3.9
Min	1950	61	42	1.2	23	8	121	21
Median	3080	81	50	1.5	32	9	126	28
Max	4590	91	70	1.7	36	16	126	33
n	9	9	9	9	7	7	4	5

SD, standard deviation; MaN, macronucleus; Infund., infundibulum.

## Data Availability

The datasets used and/or analyzed during the current study are available from the corresponding author upon reasonable request.

## Supplementary Materials

The Supplementary Materials are available online at https://www.mdpi.com/article/10.3390/life13051110/s1. Table S1: Accession numbers of the 18S rDNA gene sequences of the commensal ciliates isolated from the African millipede *Archispirostreptus gigas*; Table S2: Accession numbers of the 18S rDNA gene sequences of the ciliates used in the phylogenetic tree.

## References

[1] Albaret J.L. (1975). Étude systématique et cytologique sur les ciliés hétérotriches endocommensaux (Systematic and cytological study on heterotrichous endocommensal ciliates). Mémoires Muséum Natl. D’histoire Nat. Ser. A Zool..

[2] Gijzen H.J., Broers C.A., Barughare M., Stumm C.K. (1991). Methanogenic bacteria as endosymbionts of the ciliate Nyctotherus ovalis in the cockroach hindgut. Appl. Environ. Microbiol..

[3] Gijzen H.J., Barugahare M. (1992). Contribution of anaerobic protozoa and methanogens to hindgut metabolic activities of the American cockroach, *Periplaneta americana*. Appl. Environ. Microbiol..

[4] Gijzen H.J., van der Drift C., Barugahare M., Op den Camp H.J. (1994). Effect of Host Diet and Hindgut Microbial Composition on Cellulolytic Activity in the Hindgut of the American Cockroach, *Periplaneta americana*. Appl. Environ. Microbiol..

[5] De Graaf R.M., Ricard G., van Alen T.A., Duarte I., Dutilh B.E., Burgtorf C., Kuiper J.W.P., van der Staay G.W.M., Tielens A.G.M., Huynen M.A. (2011). The Organellar Genome and Metabolic Potential of the Hydrogen-Producing Mitochondrion of *Nyctotherus ovalis*. Mol. Biol. Evol..

[6] Balch H.E. (1967). The Cultivation of *Nyctotherus ovalis* and *Endamoeba blattae*. Science.

[7] Nelson E.C. (1943). Cultivation of *Nyctotherus cordiformis*. J. Parasitol..

[8] Lom J. (1956). Experiments with the cultivation of our three species of the genus *Nyctotherus* and of *Balantidium entozoon* and *B. coli*. Věst. Českoslov. Zool. Spol..

[9] Chen L. (1933). Züchtungsversuche an parasitischen Protozoen von *Periplaneta orientalis*. Zeitschrift. Parasitenkd..

[10] Byzov B.A., König H., Varma A. (2006). Intestinal Microbiota of Millipedes. Intestinal Microorganisms of Termites and Other Invertebrates.

[11] Šustr V., Tajovský K., Semanová S., Chroňáková A., Šimek M. (2013). The giant African millipede, *Archispirostreptus gigas* (Diplopoda: Spirostreptida), a model species for ecophysiological studies. Acta Soc. Zool. Bohem..

[12] Nardi J.B., Bee C.M., Taylor S.J. (2016). Compartmentalization of microbial communities that inhabit the hindguts of millipedes. Arthropod Struct. Dev..

[13] Taylor E.C. (1982). Role of Aerobic Microbial Populations in Cellulose Digestion by Desert Millipedes. Appl. Environ. Microbiol..

[14] Šustr V., Semanová S., Rost-Roszkowska M.M., Tajovský K., Sosinka A., Kaszuba F. (2020). Enzymatic activities in the digestive tract of spirostreptid and spirobolid millipedes (Diplopoda: Spirostreptida and Spirobolida). Comp. Biochem. Physiol. Part B Biochem. Mol. Biol..

[15] Regensbogenova M., Kisidayova S., Michalowski T., Javorsky P., Moon-Van Der Staay S.Y., Moon-Van Der Staay G.W.M.M., Hackstein J.H.P., Mcewan N.R., Jouany J.-P., Newbold J.C. (2004). Rapid identification of rumen protozoa by restriction analysis of amplified 18S rRNA gene. Acta Protozool..

[16] Katoh K., Standley D.M. (2013). MAFFT multiple sequence alignment software version 7: Improvements in performance and usability. Mol. Biol. Evol..

[17] Hall T.A. (1999). BioEdit: A User-Friendly Biological Sequence Alignment Editor and Analysis Program for Windows 95/98/NT.

[18] Ronquist F., Teslenko M., Van Der Mark P., Ayres D.L., Darling A., Höhna S., Larget B., Liu L., Suchard M.A., Huelsenbeck J.P. (2012). MrBayes 3.2: Efficient Bayesian phylogenetic inference and model choice across a large model space. Syst. Biol..

[19] Guindon S., Dufayard J.F., Lefort V., Anisimova M., Hordijk W., Gascuel O. (2010). New algorithms and methods to estimate maximum-likelihood phylogenies: Assessing the performance of PhyML 3.0. Syst. Biol..

[20] Swofford D.L. (2003). PAUP*: Phylogenetic Analysis Using Parsimony and Other Methods, Version 4.

[21] Foissner W. (2014). An update of “basic light and scanning electron microscopic methods for taxonomic studies of ciliated protozoa”. Int. J. Syst. Evol. Microbiol..

[22] Foissner W. (1991). Basic light and scanning electron microscopic methods for taxonomic studies of ciliated protozoa. Eur. J. Protistol..

[23] Doddema H.J., Vogels G.D. (1978). Improved identification of methanogenic bacteria by fluorescence microscopy. Appl. Environ. Microbiol..

[24] Williams A.G., Coleman G.S. (1992). The Rumen Protozoa.

[25] Kišidayová S., Váradyová Z., Zeleňák I., Siroka P. (2000). Methanogenesis in rumen ciliate cultures of Entodinium caudatum and Epidinium ecaudatum after long-term cultivation in a chemically defined medium. Folia Microbiol..

[26] Hino T., Kametaka M., Kandatsu M. (1973). The cultivation of rumen oligotrich protozoa. III. White clover factors which stimulate the growth of *Entodinia*. J. Gen. Appl. Microbiol..

[27] Coleman G.S., Taylor A.E.R., Baker J.R. (1978). Rumen entodiniomorphid protozoa. Methods of Cultivating Parasites In Vitro.

[28] Horáková K., Betina V., Barátová H., Fargašová A., Frank V., Horáková K., Šturdík E. (1988). Mikroskopické metódy (Microscopic methods). Mikrobiologické Laboratórne Metódy (Microbiological Laboratory Methods).

[29] Grishagin I.V. (2015). Automatic cell counting with ImageJ. Anal. Biochem..

[30] Abramoff M.D., Magalhães P.J., Ram S.J. (2004). Image processing with ImageJ. Biophotonics Int..

[31] Kišidayová S., Váradyová Z. (2005). Effect of insulin on in vitro fermentation activity of microrganism community of rumen ciliate culture. Cell Biol. Int..

[32] Cottyn B.G., Boucque C.V. (1968). Rapid method for the gas-chromatographic determination of volatile fatty acids in rumen fluid. J. Agric. Food Chem..

[33] Chaney A.L., Marbach E.P. (1962). Modified Reagents for Determination of Urea and Ammonia. Clin. Chem..

[34] Mellenberger R.W., Satter L.D., Millett M.A., Baker A.J. (1970). An in vitro Technique for Estimating Digestibility of Treated and Untreated Wood. J. Anim. Sci..

[35] Nevel C.J., Demeyer D.I. (1979). Stoichiometry of carbohydrate fermentation and microbial growth efficiency in a continous culture of mixed rumen bacteria. Eur. J. Appl. Microbiol. Biotechnol..

[36] Ungerfeld E.M. (2015). Shifts in metabolic hydrogen sinks in the methanogenesis-inhibited ruminal fermentation: A meta-analysis. Front. Microbiol..

[37] Čársky J., Ferenčík M., Škárka B. (1981). Príprava presných, tlmivých a izotonických roztokovo (Preparation of precise, buffered and isotonic solutions). Biochemické Laboratórne Metódy (Biochemical Laboratory Methods).

[38] Bradford M.M. (1976). A rapid and sensitive method for the quantitation of microgram quantities of protein utilizing the principle of protein-dye binding. Anal. Biochem..

[39] Miller G.L. (1959). Use of Dinitrosalicylic Acid Reagent for Determination of Reducing Sugar. Anal. Chem..

[40] Bailey M.J., Biely P., Poutanen K. (1992). Interlaboratory testing of methods for assay of xylanase activity. J. Biotechnol..

[41] Béra-Maillet C., Devillard E., Cezette M., Jouany J.-P., Forano E. (2005). Xylanases and carboxymethylcellulases of the rumen protozoa *Polyplastron multivesiculatum*, *Eudiplodinium maggii*, and *Entodinium* sp. FEMS Microbiol. Lett..

[42] Miller G.L., Blum R., Glennon W.E., Burton A.L. (1960). Measurement of carboxymethylcellulase activity. Anal. Biochem..

[43] Small E.B., Marszalek D.S., Antipa G.A. (1971). A Survey of Ciliate Surface Patterns and Organelles as Revealed with Scanning Electron Microscopy. Trans. Am. Microsc. Soc..

[44] Imai S., Katsuno M., Tsunoda K. (1977). Scanning Electron Microscopy of Rumen Ciliates in Cattle. Zool. Mag..

[45] Wang Q., Zhao X. (2015). A three-dimensional phase diagram of growth-induced surface instabilities. Sci. Rep..

[46] Lynn D.H. (1991). The implications of recent descriptions of kinetid structure to the systematics of the ciliated protists. Protoplasma.

[47] De Beaurepaire Aragão B. (1912). Noticia sobre o Nyctotherus cordiformis Stein. Mem. Inst. Oswaldo Cruz..

[48] Pinto C. (1926). Anatomia e biologia dos Nyctotherus do batrachios do Brasil. Bol. Biol..

[49] Nelson E.C. (1943). Alcohol–preserved tissue–cultivation medium: Methods of preparation and use and results obtained in the cultivation of *Nyctotherus cordiformis*. Am. J. Epidemiol..

[50] Lucas C.L.T. (1928). A Study of Excystation in *Nyctotherus ovalis*: With Notes on Other Intestinal Protozoa of the Cockroach. J. Parasitol..

[51] Kudo R.R. (1936). Studies on *Nyctotherus ovalis* Leidy, with special reference to its nuclear structure. Arch. Protistenkd..

[52] Suzuki J., Kobayashi S., Yoshida N., Azuma Y., Kobayashi-Ogata N., Kartikasari D.P., Yanagawa Y., Iwata S. (2020). Phylogenetic position of *Nyctotherus teleacus* isolated from a tortoise (*Astrochelys radiata*) and its electron microscopic features. J. Vet. Med. Sci..

[53] Hoyte H.M.D. (1961). The protozoa occurring in the hind-gut of cockroaches. I. Responses to changes in environment. Parasitology.

[54] Lom J. (1955). Polysacharidové reservy nálevníků rodu *Balantidium* a *Nyctotherus*. Českoslov. Biol..

[55] Dutta G.P. (1958). The Cytoplasmic Inclusions of *Nyctotherus macropharyngeus*: Histochemical Studies. J. Cell Sci..

[56] Hoyte H.M.D. (1961). The protozoa occurring in the hind-gut of cockroaches. II. Morphology of *Nyctotherus ovalis*. Parasitology.

[57] Nakai Y., Imai S. (1989). Cytochemical Identification of Reserve Polysaccharides in Rumen Ciliates by Microspectrophotometry. Jpn. J. Parasitol..

[58] Brust H., Orzechowski S., Fettke J. (2020). Starch and Glycogen Analyses: Methods and Techniques. Biomolecules.

[59] Wakita M., Hoshino S. (1980). Physicochemical properties of a reserve polysaccharide from sheep rumen ciliates genus *Entodinium*. Comp. Biochem. Physiol. Part B Comp. Biochem..

[60] Wani A.A., Singh P., Shah M.A., Schweiggert-Weisz U., Gul K., Wani I.A. (2012). Rice Starch Diversity: Effects on Structural, Morphological, Thermal, and Physicochemical Properties-A Review. Compr. Rev. Food Sci. Food Saf..

[61] Zhao L., Pan T., Guo D., Wei C. (2018). A simple and rapid method for preparing the whole section of starchy seed to investigate the morphology and distribution of starch in different regions of seed. Plant Methods.

[62] Horváthová T., Šustr V., Chroňáková A., Semanová S., Lang K., Dietrich C., Hubáček T., Ardestani M.M., Lara A.C., Brune A. (2021). Methanogenesis in the Digestive Tracts of the Tropical Millipedes *Archispirostreptus gigas* (Diplopoda, Spirostreptidae) and *Epibolus pulchripes* (Diplopoda, Pachybolidae). Appl. Environ. Microbiol..

[63] Ellis J.E., Williams A.G., Lloyd D. (1989). Oxygen consumption by ruminal microorganisms: Protozoal and bacterial contributions. Appl. Environ. Microbiol..

[64] Ellis J.E., Mcintyre P.S., Saleh M., Williams A.G., Lloyd D. (1991). The influence of ruminal concentrations of O2 and CO2 on fermentative metabolism of the rumen entodiniomorphid ciliate *Eudiplodinium maggii*. Curr. Microbiol..

[65] Park T., Yu Z. (2018). Aerobic cultivation of anaerobic rumen protozoa, *Entodinium caudatum* and *Epidinium caudatum*. J. Microbiol. Methods.

[66] Fievez V., Piattoni F., Mbanzamihigo L., Demeyer D. (1999). Reductive Acetogenesis in the Hindgut and Attempts to its Induction in the Rumen—A Review. J. Appl. Anim. Res..

[67] Laho T., Váradyová Z., Mihaliková K., Kišidayová S. (2013). Fermentation Capacity of Fecal Microbial Inocula of Przewalski Horse, Kulan, and Chapman Zebra and Polysaccharide Hydrolytic Activities of Fecal Microbial Constituents (Ciliates and Bacteria) of Kulan and Chapman Zebra. J. Equine Vet. Sci..

[68] Kišidayová S., Váradyová Z., Pristaš P., Piknová M., Nigutová K., Petrželková K.J., Profousová I., Schovancová K., Kamler J., Modrý D. (2009). Effects of high- and low-fiber diets on fecal fermentation and fecal microbial populations of captive chimpanzees. Am. J. Primatol..

[69] Cieslak A., Váradyová Z., Kišidayová S., Szumacher-Strabel M. (2009). The effects of linoleic acid on the fermentation parameters, population density, and fatty-acid profile of two rumen ciliate cultures, *Entodinium caudatum* and *Diploplastron affine*. Acta Protozool..

[70] Ivan M., Neill L., Forster R., Alimon R., Rode L.M., Entz T. (2000). Effects of *Isotricha*, *Dasytricha*, *Entodinium*, and Total Fauna on Ruminal Fermentation and Duodenal Flow in Wethers Fed Different Diets. J. Dairy Sci..

[71] Boxma B., de Graaf R.M., van der Staay G.W.M., van Alen T.A., Ricard G., Gabaldón T., Van Hoek A.H.A.M., Der Staay S.Y.M.-V., Koopman W.J.H., Van Hellemond J.J. (2005). An anaerobic mitochondrion that produces hydrogen. Nature.

[72] Šustr V., Chroňáková A., Semanová S., Tajovský K., Šimek M. (2014). Methane production and methanogenic Archaea in the digestive tracts of millipedes (Diplopoda). PLoS ONE.

[73] Finlay B.J., Esteban G., Clarke K.J., Williams A.G., Embley T.M., Hirt R.P. (1994). Some rumen ciliates have endosymbiotic methanogens. FEMS Microbiol. Lett..

[74] Lewis W.H., Sendra K.M., Embley T.M., Esteban G.F. (2018). Morphology and Phylogeny of a New Species of Anaerobic Ciliate, *Trimyema finlayi* n. sp., with Endosymbiotic Methanogens. Front. Microbiol..

[75] Vogels G.D., Hoppe W.F., Stumm C.K. (1980). Association of methanogenic bacteria with rumen ciliates. Appl. Environ. Microbiol..

[76] Tóthová T., Piknová M., Kišidayová S., Javorský P., Pristaš P. (2008). Distinctive archaebacterial species associated with anaerobic rumen protozoan Entodinium caudatum. Folia Microbiol..

[77] Stumm C.K., Zwart K.B. (1986). Symbiosis of protozoa with hydrogen-utilizing methanogens. Microbiol. Sci..

[78] Goosen N.K., Horemans A.M.C., Hillebrand S.J.W., Stumm C.K., Vogels G.D. (1988). Cultivation of the sapropelic ciliate *Plagiopyla nasuta* Stein and isolation of the endosymbiont *Methanobacterium formicicum*. Arch. Microbiol..

[79] Regensbogenova M., Michalowski T., Newbold C., McEwan N., Javorsky P., Kišidayová S., Hackstein J., Pristaš P. (2004). A re-appraisal of the diversity of the methanogens associated with the rumen ciliates. FEMS Microbiol. Lett..

[80] Nunez F.S., Crawford C.S. (1976). Digestive enzymes of the desert millipede *Orthoporus ornatus* (Girard) (Diplopoda: Spirostreptidae). Comp. Biochem. Physiol. Part A Physiol..

[81] Profousová I., Mihaliková K., Laho T., Váradyová Z., Petrželková K.J., Modrý D., Kišidayová S. (2011). The ciliate, *Troglodytella abrassarti*, contributes to polysaccharide hydrolytic activities in the chimpanzee colon. Folia Microbiol..

[82] Kišidayová S., Pristaš P., Zimovčáková M., Blanár Wencelová M., Homol’ová L., Mihaliková K., Čobanová K., Grešáková Ľ., Váradyová Z. (2018). The effects of high dose of two manganese supplements (organic and inorganic) on the rumen microbial ecosystem. PLoS ONE.

[83] González G., Murphy C.M., Belén J., Sudarshana P., Nageswara-Rao M., Soneji J.R. (2012). Direct and Indirect Effects of Millipedes on the Decay of Litter of Varying Lignin Content. Tropical Forests.

[84] Demeyer D.I., De Graeve K. (1991). Differences in Stoichiometry Between Rumen and Hindgut Fermentation. Adv. Anim. Physiol. Anim. Nutr..

[85] Sardar P., Šustr V., Chroňáková A., Lorenc F. (2022). Metatranscriptomic holobiont analysis of carbohydrate-active enzymes in the millipede *Telodeinopus aoutii* (Diplopoda, Spirostreptida). Front. Ecol. Evol..

[86] Hogan M.E., Slaytor M., O’Brien R.W. (1985). Transport of volatile fatty acids across the hindgut of the cockroach *Panesthia cribrata* Saussure and the termite, *Mastotermes darwiniensis* Froggatt. J. Insect Physiol..

[87] Leidy J. (1853). Some Observations on *Nematoidea Imperfecta*, and Descriptions of Three Parasitic Infusoriae. Trans. Am. Philos. Soc..

[88] Leidy J. (1849). *Nyctotherus*, a new genus of Polygastrica, allied to Plescoma. Proc. Acad. Nat. Sci. USA.

[89] Savin M.B. (1931). *Nyctotherus velox* and *Endolimax* sp. with Remarks on Other Prozozoan Parasites Found in the Millipede, *Spirobolus marginatus*. Master’s Thesis.

[90] van Hoek A.H., van Alen T.A., Sprakel V.S., Hackstein J.H., Vogels G.D. (1998). Evolution of anaerobic ciliates from the gastrointestinal tract: Phylogenetic analysis of the ribosomal repeat from *Nyctotherus ovalis* and its relatives. Mol. Biol. Evol..

[91] Lynn D.H., Wright A.-D.G. (2013). Biodiversity and Molecular Phylogeny of Australian Clevelandella Species (Class Armophorea, Order Clevelandellida, Family Clevelandellidae), Intestinal Endosymbiotic Ciliates in the Wood-Feeding Roach *Panesthia cribrata* Saussure, 1864. J. Eukaryot. Microbiol..

[92] Li M., Hu G., Li C., Zhao W., Zou H., Li W., Wu S.-G., Wang G.-T., Ponce-Gordo F. (2020). Morphological and molecular characterization of a new ciliate *Nyctotheroides grimi* n. sp. (Armophorea, Clevelandellida) from Chinese frogs. Acta Trop..

[93] Pecina L., Vďačný P. (2020). Two New Endozoic Ciliates, *Clevelandella lynni* sp. n. and *Nyctotherus galerus* sp. n., Isolated from the Hindgut of the Wood-feeding Cockroach *Panesthia angustipennis* (Illiger, 1801). J. Eukaryot. Microbiol..

[94] Paiva T.D.S., Borges B.D.N., Silva-Neto I.D.D. (2013). Phylogenetic study of Class Armophorea (Alveolata, Ciliophora) based on 18S-rDNA data. Genet. Mol. Biol..

[95] Pecina L., Vďačný P. (2020). Morphological versus molecular delimitation of ciliate species: A case study of the family Clevelandellidae (Protista, Ciliophora, Armophorea). Eur. J. Taxon..

[96] Affa’a F.M., Hickey D.A., Strüder-Kypke M., Lynn D.H. (2004). Phylogenetic position of species in the genera *Anoplophrya*, *Plagiotoma*, and *Nyctotheroides* (*Phylum ciliophora*), endosymbiotic ciliates of annelids and anurans. J. Eukaryot. Microbiol..

