# Supplementary material for: Some Aspects of the Physiology of the Nyctotherus velox, a Commensal Ciliated Protozoon Taken from the Hindgut of the Tropical Millipede Archispirostreptus gigas
Source: Life (Basel). 2023 Apr 29;13(5):1110. doi: 10.3390/life13051110 (PMC10223032; doi:10.3390/life13051110)
Supplement: Supplementary file 1 [file life-13-01110-s001.zip › life-2244353-supplementary.pdf]

Supplementary material

Supplementary Table S1: Accession numbers of the 18S rDNA gene sequences of the commensal ciliates isolated from the African millipede *Archispirostreptus gigas*.

Species name	Isolate label	GenBank Accession number	Collection year	Country of origin of the host	Locality	Host Organism	Source
Nyctotherus velox (Leidy, 1849)	2K_AG	OQ474962	2012	Tanzania	Laboratory breeding, Czech Republic	*Archispirostreptus gigas* (Peters, 1868)	Ciliate culture from hindgut
Nyctotherus velox (Leidy, 1849)	3K_AG	OQ474963	2012	Tanzania	Laboratory breeding, Czech Republic	*Archispirostreptus gigas* (Peters, 1868)	Ciliate culture from hindgut
Nyctotherus velox (Leidy, 1849)	4K_AG	OQ474964	2012	Tanzania	Laboratory breeding, Czech Republic	*Archispirostreptus gigas* (Peters, 1868)	Ciliate culture from hindgut
Nyctotherus velox (Leidy, 1849)	6K_AG	OQ474965	2012	Tanzania	Laboratory breeding, Czech Republic	*Archispirostreptus gigas* (Peters, 1868)	Ciliate culture from hindgut
Nyctotherus velox (Leidy, 1849)	7K_AG	OQ474966	2012	Tanzania	Laboratory breeding, Czech Republic	*Archispirostreptus gigas* (Peters, 1868)	Ciliate culture from hindgut
Nyctotherus velox (Leidy, 1849)	8K_AG	OQ474967	2012	Tanzania	Laboratory breeding, Czech Republic	*Archispirostreptus gigas* (Peters, 1868)	Ciliate culture from hindgut
Nyctotherus velox (Leidy, 1849)	9K_AG	OQ474968	2012	Tanzania	Laboratory breeding, Czech Republic	*Archispirostreptus gigas* (Peters, 1868)	Ciliate culture from hindgut
Nyctotherus velox (Leidy, 1849)	10K_AG	OQ474969	2012	Tanzania	Laboratory breeding, Czech Republic	*Archispirostreptus gigas* (Peters, 1868)	Ciliate culture from hindgut
Nyctotherus archispirostreptae n. sp.	3E_AG	OQ474960	2012	Tanzania	Laboratory breeding, Czech Republic	*Archispirostreptus gigas* (Peters, 1868)	Hindgut content
Nyctotherus archispirostreptae n. sp.	8E_AG	OQ474959	2012	Tanzania	Laboratory breeding, Czech Republic	*Archispirostreptus gigas* (Peters, 1868)	Hindgut content
Nyctotherus velox (Leidy, 1849)	11E_AG	OQ474961	2012	Tanzania	Laboratory breeding, Czech Republic	*Archispirostreptus gigas* (Peters, 1868)	Hindgut content

Supplementary Table S2: Accession numbers of the 18S rDNA gene sequences of the ciliates used in the phylogenetic tree

Species name	GenBank Accession number
Nyctotherus velox	AJ006713.1
Anteclevelandella constricta	MT675973.1
Clevelandella constricta	KC139713.1
Clevelandella hastula	MT675987.1
Clevelandella lynni	MN966414.1
Clevelandella nipponensis	KC139714.1
Clevelandella panesthiae	MT675990.1
Clevelandella parapanesthiae	MT675998.1
Metopus fuscus	KF607083.1
Metopus hasei	MH086817.1
Metopus minor	MH086822.1
Metopus setosus	KY855536.1
Nyctotheroides cordiformis	MK084748.1
Nyctotheroides deslierresae	AF145353.1
Nyctotheroides grimi	MK087045.1
Nyctotheroides hubeiensis	KT247989.1
Nyctotheroides parvus	AF145352.1
Nyctotheroides pyriformis	KX010599.1
Nyctotherus galerus	MN966415.1
Nyctotherus ovalis	AJ222678.1
Nyctotherus sp.	KC139720.1
Nyctotherus sp.	KC139721.1
Paraclevelandia brevis	MT676006.1
Sicuophora multigranularis	MH301103.1
